# Metasurfaces as Energy Valves for Sustainable Energy Management

**DOI:** 10.3390/mi13101769

**Published:** 2022-10-18

**Authors:** Yoshiaki Nishijima, Syunya Kimura, Yu Takeshima, Saulius Juodkazis

**Affiliations:** 1 Department of Electrical and Computer Engineering, Graduate School of Engineering, Yokohama National University, 79-5 Tokiwadai, Hodogaya-ku, Yokohama 240-8501, Japan; 2 Institute of Advanced Sciences, Yokohama National University, 79-5 Tokiwadai, Hodogaya-ku, Yokohama 240-8501, Japan; 3 Optical Sciences Centre and ARC Training Centre in Surface Engineering for Advanced Materials (SEAM), School of Science, Swinburne University of Technology, Melbourne, VIC 3122, Australia; 4 WRH Program International Research Frontiers Initiative (IRFI) Tokyo Institute of Technology, Nagatsuta-cho, Midori-ku, Yokohama 226-8503, Japan

**Keywords:** mid-infrared plasmonics, thermal radiation, transparent metasurfaces

## Abstract

Control of light absorption and transmission by metal–insulator–metal (MIM) metasurfaces are promising for applications in optical windows. This study shows the realization of photo-thermal energy conversion for radiative cooling by MIM metasurfaces with thin metal substrate and Indium–Tin–Oxide (ITO). High transparency of ITO at visible wavelengths and high absorption at mid-infrared wavelengths were realized for future applications of efficient cooling or heating applicable for living and working spaces. The MIM (ITO/CaF_2_/ITO) metasurface was patterned with low-resolution photo-lithography as a demonstration of further simplification and possible scalability of the patterning for practical window applications.

## 1. Introduction

The nano-/micro-materials that show plasmon resonances have attracted interest in the control of light-matter interactions at the resonance wavelengths. Conventional plasmonic materials such as nanoparticles and nanodiscs (referred to as scattering/reflection type plasmonic materials) and metal hole arrays (transparent plasmonic materials) are increasingly utilized in surface-enhanced spectroscopies, active nanophotonics (nano-lasing), bio-sensing, and invisibility cloaking among a much broader field [1,2,3]. Recently, control of optical absorption has been sought after as a key technology for high-efficiency energy harvesting. For this purpose, an absorption type of plasmonic material, the so-called plasmon perfect absorber or metal–insulator–metal (MIM) metasurfaces, which are increasingly investigated for applications of efficient photo-thermal energy conversion [4,5,6,7,8,9,10,11,12,13,14,15,16,17,18,19,20,21,22,23,24,25]. Especially appealing is the sky radiator application, a solution for the cooling room through the optical window by exploiting the high transparency of air at the 7–14 μm mid-IR spectral window. The light absorption by MIM metasurfaces is used for cooling. When the top nano-/micro-discs of the MIM metasurface are in resonance with the incident light, this plasmonic resonance is coupled with the induced plasmonic resonance of the bottom metal layers of the MIM structure. At the resonance, the incident light is localized in the insulator layer (of MIM) and decays by absorption loss in the metals and insulator layers. Therefore, the optical response of MIM metasurfaces depends on the disc diameter and metal and insulator materials as shown in our previous studies of the extensive search of optimized geometric parameters [23].

The principle of photo-thermal energy conversion is founded on Kirchhoff’s law of thermal radiation. This law determines equivalence between the thermal radiation ϵ and absorption α efficiencies. Therefore, MIM metasurfaces that perform as perfect absorbers are also perfect radiators. Typical metasurfaces for absorption control are made using an MIM layout of a metal base plate, insulator, and metal nanostructures. A thick ∼200 nm metal base plate prevents optical transmission at any wavelength.

**Figure 1:**
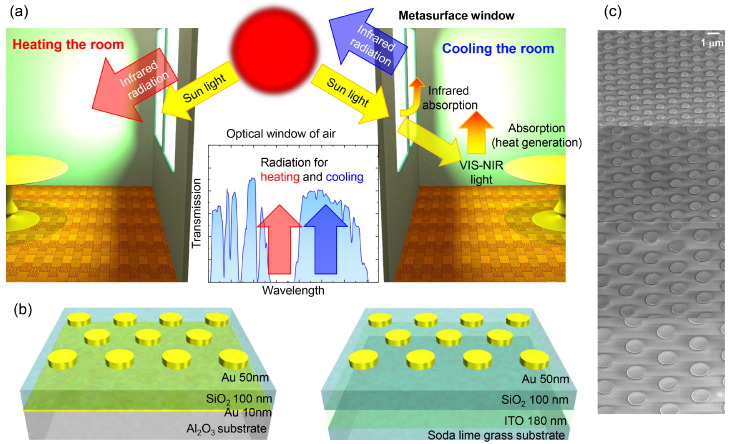
(**a**) Schematic illustration of passive control of energy flow in visible and near-IR transparency windows for the radiative cooling (right) and MIR absorptive metasurface for radiative heating (left). (**b**) Design of required metasurfaces investigated in this study: transparency at visible and absorbance (emittance) at the MIR spectral range for energy-valve windows. (**c**) Scanning electron microscopy (SEM) images of metasurfaces made of discs with 700 nm, 1000 nm, 1300 nm, 1700 nm diameters; SEM images were taken at tilted 45° angle.

The concept of radiation cooling is based on the thermal energy generated in the room to be emitted into the atmospheric IR transparency window of air. The MIM metasurface can serve as the emitter. In these applications, metasurfaces have to be transparent at visible wavelengths, ideally applicable to surfaces of buildings (or windows) as paint coatings. Especially for optical window applications, it is important to have properties of transparency at visible wavelengths while keeping a blocking (absorption and reflection) capability at the mid-infrared (MIR) spectral range. Another useful and complimentary functionality of thermal management in a building would be the realization of radiative heating by enhancing the greenhouse effect inside the room via control of the radiation wavelength at the air absorption band (rather than transmission used for radiative cooling). The concept of technology to control the temperature of ambient livable space without consuming energy can contribute to intelligent buildings and agriculture. Raman et al. estimated that by reducing room temperature by 5 °C, the energy savings reach 40 W/m^2^ [26]. Specifically, if we could create an environment that stably supplies crops regardless of global climate change, it would be a key technology to realize and promote a “zero hunger” in Sustainable Development Goals (SDG) [27]. Such technology has the promise to end hunger, achieve food security, improve nutrition, and promote sustainable agriculture. Hence, it is very important to realize the control of transmittance *T*, reflectance *R*, and absorbance *A* over the visible wavelength transparency (to sunlight) and at the mid-IR spectral window. Metasurfaces are very promising materials enabling easy control of their absorption and radiation wavelength via the geometrical structure of MIM structures. Ideally, by control of heating and cooling with passive devices, it should be possible to create comfortable conditions in the room without (at a reduced) electric energy consumption. In agriculture, passive control of greenhouse conditions is required for stable crop harvesting. The need for such technology will be exacerbated due to climate change. The concept of radiative heating and cooling by the metasurface window is shown in Figure 1a with a transmission valve metasurface shown in Figure 1b. The proposed metasurfaces will be used as optical windows, which are the main gate for heat transfer between the inside (room) and outside ambiance. The visible light that enters the room warms it up via light absorption. Then, the window itself is also warmed up. The radiation direction and wavelength of the designed metasurfaces will control whether the radiation energy is held inside the room (heating) or can be dispatched outside by radiation (cooling).

In this study we demonstrate MIM structures for combined functionality of visible wavelength transparency with MIR absorption for applications in optical windows via the following designs based on: (1) thin Au film (∼10 nm) for metal ground plate (thin-Au) and (2) Indium–Tin–Oxide (ITO) as the metal ground plate [28,29,30,31,32,33,34,35,36,37,38,39,40,41]. The first design is adopted for windows with radiative cooling and the second for heating a room via the greenhouse effect.

## 2. Experimental Procedures and Modeling

### 2.1. Fabrication of Transparent Metasurfaces

Metasurfaces were fabricated via standard electron beam lithography (EBL) protocols. Both thin-Au and ITO substrates were prepared via the following route. First, the thin-Au film has vacuum evaporated on a sapphire Al_2_O_3_ substrate with 2 nm of Cr as the adhesion layer. Then, a 100 nm SiO_2_ film was deposited using electron beam (EB)-evaporation without any adhesion layer. ITO substrates were commercially acquired with 180 nm thickness. They had ∼6 Ω/sq. sheet resistance, which showed 10${}^{5}$ to 10${}^{6}\;(\Omega$.cm)^−1^ conductivity on the soda-lime glass substrate. A film of 100 nm of SiO_2_ was deposited using EB-evaporation without an adhesion layer.

For both substrates (Figure 1b), EBL was used to define patterns of nano-discs from 300 nm to 2000 nm diameters. Then, 50 nm of Au was evaporated with 2 nm Cr. Lift-off was performed by dipping it into an organic solvent. N-Methyl-2-pyrrolidone (NMP) was heated to 100 °C until the metal-ion-resist layer floated. Then, samples were washed in acetone and methanol and dried by air blowing, resulting in the final transparent MIM windows (Figure 1b) used for characterization. Scanning electron microscopy (SEM) was used for structural characterization of MIM metasurfaces (Figure 1c).

### 2.2. Optical Characterization

Optical transmission and reflection spectra of fabricated windows (Figure 1b) were measured using microscopy-based spectroscopic techniques. At visible wavelengths, an upright microscope (Olympus BX-63) with a combined spectrometer and cooled CCD (SP2150 and PIXIS 256E, Teledyne Princeton instruments) was implemented. For MIR wavelengths, FT-IR (FTIR-6600, JASCO) with microscope accessory (IRT-1000, JASCO) was used. The reference of transmission and reflection was a broad-band reflection mirror (100% from 400 nm to 2000 nm) and Au-mirror (98% at MIR wavelengths). Air ambient was used for transmission measurements [25].

### 2.3. FDTD Simulation of MIM Properties

Finite difference time domain (FDTD) simulations were performed with a homemade workstation with a Dual CPU (AMD EPYC 7302 16 core × 2) and 512 GB of DDR4 memories. For the spectrally advanced modeling, high accuracy simulations were performed to reveal detailed spectral features. Commercial software FDTD solution (ANSYS/Lumerical) was used for numerical modeling. The simulation region was set using double period nano/micro-disc structure along the lateral X- and Y-directions of the triangular lattice arrangement. The periodic boundary conditions were set in-plane (X- and Y-directions) and the perfect matching layer (PML) in the Z-direction. The mesh was set to “non-uniform” with a mesh accuracy of two (typical value for time efficient calculations). For a wide wavelength region from the visible to MIR, Palik’s optical permittivity data were used for Au, Cr, SiO_2_, Al_2_O_3_. For the ITO properties, which were difficult to measure experimentally over the entire vis-MIR spectral range, we set up a model based on the 3D conductivity and Drude plasma models described next. For the Drude’s free electron model in two equivalent presentations [42]:

(1) $$\varepsilon \left(\omega \right)=\varepsilon_{\mathrm{DC}}+i\frac{\sigma_{\mathrm{DC}}}{\omega \varepsilon_{0}},$$

(2) $$\varepsilon \left(\omega \right)=\varepsilon_{\infty }-\frac{\omega_{p}}{\omega^{2}+i\omega \Gamma },$$

where ε(ω) defines the frequency dependence of permittivity, $\varepsilon_{\mathrm{DC}}$ is the permittivity of ITO at the direct current (DC) conditions, σ is the conductivity, $\varepsilon_{0}$ is the permittivity of the vacuum, $\varepsilon_{\infty }$ is the permittivity at high frequency, $\omega_{P}$ is the plasma frequency, and Γ is the damping constant. Wolf et al. [43] analyzed ITO films using the Drude model and found the parameters for the sputtered films to follow $\varepsilon_{\infty }=4.4$, $\omega_{P}=3.36\times 10^{15}$ rad/s, $\Gamma =1.05\times 10^{14}$ 1/s.

The 3D conductivity model can be used for materials that have low losses. A simple formula for conductivity at direct current (DC) conditions can be derived to define the model from DC measurements of refractive index and conductivity. However, if the material has a frequency (wavelength) dependence on electrical conductivity, it is evident that a different model has to be applied for simulations. If free electric charges can follow the AC frequency, the 3D conductive model becomes a suitable approximation. However, another model is required if the response of charges to a high frequency is not instantaneous. The Drude model is promising for modeling the free electron behavior in noble metals. It was successfully adapted for plasmonic metals Au, Ag, and Cu. The relationship between parameters of the 3D conductivity and Drude-plasma can be combined as:

(3) $$\sigma_{DC}=\left(\frac{N}{V}\right)\left(\frac{e^{2}\tau }{m^{*}}\right)=de\mu =\tau \omega_{p}^{2}$$

where *d* is the density of electrons N/V (i.e., Number/Volume), μ is their mobility, and m${}^{*}$ is the effective mass. In the Drude model of free electrons, τ is the relaxation time with τ=1/Γ. ITO films that have a high carrier (electron) density and high mobility can be described by the high $\omega_{P}$, large permittivity, and longer relaxation time τ. In optical frequency, the Drude model should be applied for the optical permittivity of ITO film. However, most of the articles report the DC conductivity of ITO. Therefore, it is important to connect both models and here we discuss both models.

## 3. Results and Discussion

### 3.1. Thin Au as a Metal Base in MIM Metasurface

**Figure 2:**
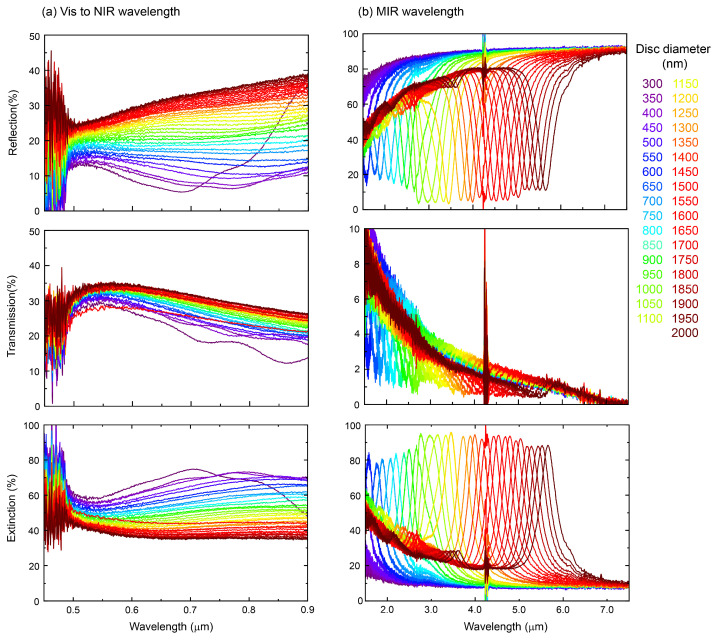
Experimental spectra. Optical reflection, transmission, and extinction spectra of thin-Au metasurfaces (Figure 1b) from visible to MIR wavelengths. (**a**) Visible to near infrared wavelength, (**b**) MIR wavelength. Thicknesses of layers in the MIM structure: Au-disc of 50 nm, SiO_2_ of 100 nm, and Au film of 10 nm (with a 2 nm of Cr adhesion layer); substrate was 0.3 mm sapphire. The period-to-diameter ratio was two. A spectrally narrow anti-reflective band (R→0) at IR wavelengths with simultaneous low transmission (T→0) marks the position of strong absorption (an expected strong thermal emission for radiative cooling).

Figure 2 shows the optical reflection, transmission, and extinction spectra within visible to near-IR and near-IR to MIR wavelength regions, respectively. The extinction Ext. (normalized) is defined as Ext.=1−(R+T). A low reflectance *R* was observed for the thin-Au MIM samples. The reflective properties of the metasurface can be explained by coupling between the plasmon resonance of metal nanodiscs and induced plasmon resonance of the metal ground plate. If the thickness of the metal ground plate becomes thinner than the skin depth in the metal (at that wavelength), the induced plasmon resonance at the Au-SiO_2_ interface is expected. Then, a forward re-radiation is expected from the metasurface under such conditions. This feature was recognizable in the transmittance *T* spectra as well. The transmission was well suppressed to less than T<10% and the reflectance spectra show a strong anti-reflection property (Figure 2). At the visible wavelengths region, transmission became less than 40% in air.

Au has a $\omega_{P}$ of 13.8 $\times 10^{15}$ rad/s, which corresponds to the 136 nm wavelength. When the frequency of light becomes larger than the $\omega_{P}$, the free electron cannot follow oscillations of the E-field of light, and metal becomes endowed with the dielectric-like property. The Au, Ag, and Cu have similar $\omega_{P}$ values at 13.8, 14.0, 13.4 ×$10^{15}$ rad/s, respectively, and at the visible spectral range spectral (color) differences in *R* and *T* of those metals mainly comes from differences in the damping constants. When the damping constant Γ became smaller (Au 1.07, Ag 0.322, Cu 1.45 ×$10^{14}$ 1/s), the high reflectance region extended to the $\omega_{P}$. Therefore, the reflectance of Au and Cu at visible wavelengths starts to decline in the red-to-near-IR wavelength range, and causes an increase in visible transmission. It would be expected that for Cu, a high transmission region would extend until longer wavelengths than for Au. However, due to the propensity for oxidation of Ag and Cu, it is challenging to fabricate pure metal (not-oxidized) stable thin metal films. Therefore, Au is the most promising material for applying a thin ∼10 nm thickness film for the required optical transmission. However, Au lacks transparency for the practical application as a window for the visible spectral range. For the window application, it is imperative to have transmissions higher than 70%.

**Figure 3:**
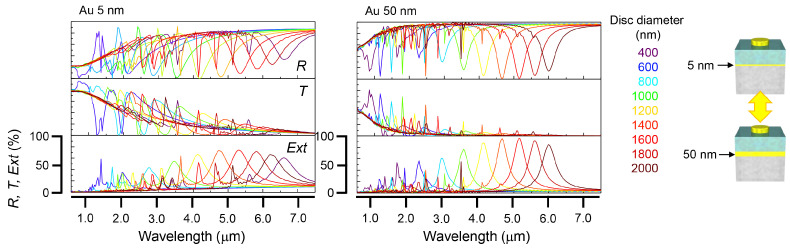
Numerical simulations. Influence of thickness of the bottom mirror in MIM. FDTD simulations for different thickness thin-Au MIM metasurface without adhesion layers.

To identify the origin of optical losses, we performed FDTD simulations for different adhesion layers of Cr, Ti, and Au thicknesses. The results are shown in Figure 3 and Figure S1. When there was no adhesion layer (Figure S1), a film of 10 nm of gold kept the overall transparency above 80% at visible wavelengths. When the adhesion layer of 5 nm Cr or Ti was added between the metal and dielectric layers, the transmittance *T* drastically decreased to less than 40%. The effect of Au ground plate thickness was simulated as shown in Figure 3 for all MIM metasurfaces with different nano-disc diameters. Interestingly, with an increasing thickness of Au, the transmission was still kept above 40%; even for the 30 nm Au film, T≈80% in the visible wavelength range. Specific to the permittivity of Au at that spectral part, a decrease was present in the Drude and Lorenz expression at around 50 nm thickness. Therefore, there is a high transparency with small absorption and reflection at those wavelengths.

Following the established trend in earlier experimental studies, it is often argued that 2 nm of Cr has enough adhesion strength for successful lift-off of metasurfaces. Thinner than 10 nm Au is expected to form an island film rather than a uniform coating. Therefore, 2 nm of Cr and 10 nm Au define the lowest limitation for experimental fabrication of a metal nano-film in MIM metasurfaces. Consequently, the transparency of 40% obtained in experiments is almost the limitation for Au as the ground plate in MIM metasurfaces. Figure S2a shows the optical permittivity of Au, Cr, and Ti obtained from Palik’s database and used in FDTD simulations of conductivity and Drude models, which were used in FDTD simulations.

### 3.2. ITO as a Metal Base in MIM Metasurface

**Figure 4:**
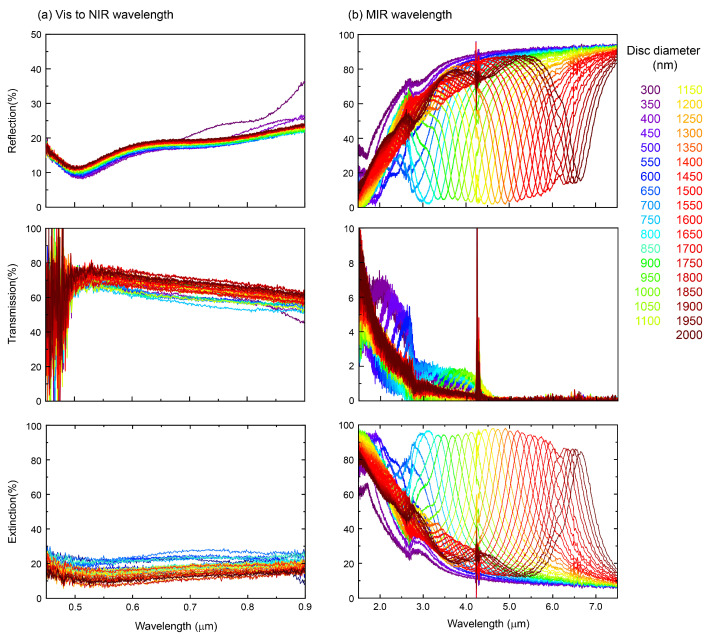
Experimental spectra. Optical reflection, transmission, and extinction spectra of ITO-base MIM metasurfaces made on a soda lime glass: visible to near-IR (**a**) and MIR wavelengths (**b**) range.

Due to the limitation of Au discussed above, it is imperative to use a transparent conductive oxide (TCO) material such as ITO, FTO (F-doped SnO_2_), AZO (Al-doped ZnO), and the like. ITO is one of the most popular and commonly used TCOs. ITO has become an essential material for the recent all-dielectric metasurfaces. The optical properties of the metasurface, with ITO as the ground plate, are shown in Figure 4. This shows a possibility to realize 80% transparency at visible wavelengths. ITO was sputtered onto a soda lime glass plate, and there was no transparency at longer than 4 μm wavelengths due to the absorption of the glass substrate. However, the plasmon resonance assists the frequency selective anti-reflective performance (absorption) at the resonance wavelengths. The reflection spectra show the anti-reflection resonance band similar to the Au ground plate in MIM metamaterials discussed previously. It is highly expected that selective thermal radiation at that MIR wavelength can be utilized for the greenhouse effect, i.e., a window facilitating the warming up inside the room. Air conditions in the room, such as humidity and CO_2_ concentration are important to the efficient absorption at MIR wavelengths, which can also be facilitated by hydroxyl-OH band absorption at the encountered surfaces: paints on the floor, walls, and furniture.

**Figure 5:**
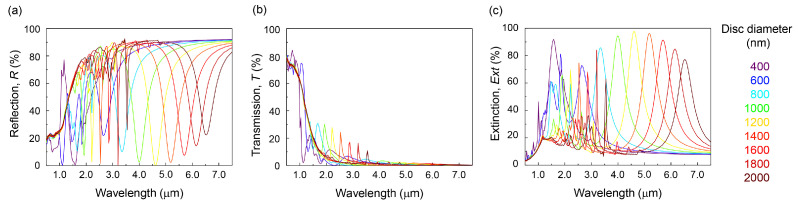
Numerical simulations. (**a**) Reflectance *R*, (**b**) Transmittance *T*, and (**c**) Extinction Ext=1−(R+T) spectra. FDTD simulations for ITO metasurfaces with the plasma model with $\varepsilon_{\infty }$ = 4.4, $\omega_{P}$ = 3.36 × 10${}^{15}$ rad/s, Γ = 1.05 × 10${}^{14}$ 1/s.

Further insights into the expected optical performance of ITO-based MIM metasurfaces can be gained from numerical modeling. For the FDTD simulation, a model of ITO permittivity is essential. In most TCO works, conductivity and refractive index are discussed. However, the conductivity itself is not a constant to the AC conditions encountered in the optical applications, i.e., the high frequency of light is not represented by the DC conductivity. Therefore, it is important to consider the Drude model, which is well established for modeling metallic response or free electron behavior at high frequencies. The model incorporating the conductive and Drude plasma response (Equation (3)) was used for FDTD simulations of reflectance *R*, transmittance *T*, and extinction (1−(R+T)), which are summarized in Figure 5. The optical properties of metasurfaces would be expected to contain the absorbance *A* and scattering *S* in the (1−(R+T)) dependence [44]. The main plasmonic absorption band appeared from 2.0 to 7.0 μm and was relatively broad. At approximately twice the shorter wavelength (than the plasmonic band), a narrow, sharp band was recognizable (Figure 5). This band originates from the lattice mode (of a phonon nature) [45,46]. The lattice mode is mainly due to scattering rather than absorption. Therefore, lattice mode is not contributing efficiently to the thermal radiation. As mentioned before, the conductive model alone cannot reproduce the experimental results well, especially in the range of anti-reflective resonances, and is limited to the 2–4 μm spectral window. Transmission in the entire modeled wavelength region visible-to-MIR became almost zero.

By using the Drude model, the FDTD results matched the experimental data well. Figures S4 and S5 show various parameter dependencies set for the both models: (1) conductive (Figure S4) and plasma (Figure S5). In the transparent conductive films, $\omega_{P}$ increases when the conductivity σ is larger by increasing the carrier density *d*. Therefore, the absorption α becomes stronger, and the transparency decreases. Therefore, the mobility μ increases the conductivity σ (see scaling shown in Equation (3)) yielding a good match with the experimental observation.

In previous experiments and simulations, it was clearly shown that scattering and absorption cross-sections $\sigma_{sca}$ and $\sigma_{abs}$, respectively, contribute differently to the thermal radiation [44]. In the system of Au back plate and Au nanodisc MIM metasurfaces, the perfect anti-reflection condition *R* = 0 always takes place when $\sigma_{sca}=\sigma_{abs}$. At this condition, thermal radiation efficiency dropped below 75%, which is less than that expected from Kirchhoff’s law, when considering the energy conservation condition when all extinction is due to the absorption losses A=1−(R+T). It was also demonstrated that an increase of $\sigma_{abs}$ rather $\sigma_{sca}$ favors the perfect anti-reflective condition, which was achieved by adding a thicker Cr layer up to 50 nm. This caused near-perfect absorption and radiation. Of course, the Cr layer cannot be used in the ITO system due to the high absorption of Cr. However, it was noticed that ITO has a higher absorption loss than Au. For the set of ITO Drude parameters, $\varepsilon_{\infty }$ = 4.4, $\omega_{P}$ = 3.36 × 10${}^{15}$ rad/s, $\Gamma =1.05\times 10^{14}$ 1/s, the 1200 nm diameter discs correspond to the near-perfect anti-reflection (hence perfect absorber) conditions. The ratio $\sigma_{abs}/\sigma_{sca}=1.14$ was obtained from FDTD simulations. This value was found weakly dependent on the $\omega_{P}$ than Γ. When Γ decreased, $\sigma_{sca}$ increased due to smaller absorption losses in ITO. Ishibashi et al. demonstrated that control of the electronic conductivity of ITO films can be achieved via the sputtering temperature and bias voltage [47]. Therefore, further refinement and optimization can be made to maximize the thermal radiation efficiency. Furthermore, fabrication of nanodiscs out of ITO is prospective for further improvement of radiation at MIR and high transparency at visible spectral ranges, respectively.

In the case of the Au base plate in MIM, the transmittance was about 40%. If the thickness of Au is less than 5 nm, an island structure will be formed, and the film will not be uniform. This is why 5 nm Au cannot serve as the base plate in MIM. However, on a substrate such as mica, a flat curtain can be obtained at the atomic level of Au [48], so it is thought that further thinning of Au can be realized. In the case of Au, it is necessary to place about 4 nm of Cr as an adhesive layer for each layer of dielectric, which causes significant losses in light transmission. In addition, the practical requirement for Au to have a film thickness of ∼10 nm will also reduce visible light’s transmittance.

Due to the extension of plasmon resonance into MIR wavelengths, transmission and reflection spectra varied with the disc diameter as shown in Figure 5 and Figure S4. The smaller disc diameters reduced transmittance at visible wavelengths. On the other hand, the optical properties of ITO show that the resonance wavelength shift towards the longer wavelengths because of lower plasma frequency $\omega_{P}$. Au has a direct absorption band at visible wavelengths, but ITO has a wide bandgap around 3 eV. Therefore, the effects of reduced *T* extending into the visible wavelength region is smaller than that for the thin-Au mirror MIM metasurfaces.

Let us further explore which transparent substrates are most promising for control of R&T at the visible and MIR spectral window. The optical transmission window of sapphire reaches 5 μm in IR, while the absorbance of soda lime glass starts at a wavelength shorter than 2.8 μm. This absorption limitation appeared in the transmission spectra in MIR. If there is no absorption by the substrate, it would be expected to observe the optical window effect for radiative cooling at the MIR wavelengths. Moreover, the sapphire substrate can be helpful in controlling the optical absorption at MIR. If we need a transparency window to extend to the longer wavelengths, CaF_2_, MgF_2_, LaF_3_ can be utilized as they reach the 7.5–10 μm cut-off wavelengths. Quartz or fused silica are not transparent at the MIR range and strongly absorb in that spectral range. For this study—the smart applications of windows for buildings—we need to block the near-IR–MIR light that is the heating source for the room. Hence, soda lime glass is better for that purpose. However, due to reciprocity, the heat generated inside the room is not left by radiation through the window (it is absorbed and reflected, causing the greenhouse effect).

Figure S5 shows comprehensive simulation results for $\varepsilon_{\infty }$ = 4.4, $\omega_{P}$ was changing from $1.0\times 10^{14}$ to $1.0\times 10^{16}$ rad/s, Γ is from $1.0\times 10^{14}$ to $1.0\times 10^{16}$ 1/s. This presents an analysis of the effect of $\omega_{P}$ and Γ on resonances due to the ground plate. The condition $\omega_{P}\geq 10^{15}$ is required to obtain an MIM metasurface with optical properties beneficial for radiative cooling and thermal emitters. Furthermore, a smaller Γ is necessary for the large amplitude modulation of *T* and *R*. If the electron conductivity is too small, which means smaller $\omega_{P}$ and larger Γ, it is difficult to form the mirror image induced by plasmon resonance on the surface of ITO due to the ohmic losses. Therefore, optical properties become similar to the nanodisc structure made out of dielectric material. The FDTD simulations show that a separation between nanodiscs and metasurfaces as well as conductivity larger than $\sigma =1\times 10^{5}\;\Omega$.cm^−1^ are required to obtain metasurface properties extending from visible to the MIR spectral region [49,50]. It is worth noting that even with a lower conductivity σ at 10${}^{4}\;\Omega$.cm^−1^, larger nanodiscs, longer wavelengths, and optical properties of metasurfaces become recognizable. This is due to the frequency dependence of the materials parameter in Equation (1), where the imaginary part $\varepsilon_{2}$ becomes a more significant following trend for a larger σ. With increasing σ, the absorption of ITO also becomes stronger and the transmittance *T* becomes markedly reduced.

### 3.3. Large-Area Fabrication for Radiation Control (an Optical Window)

It is often discussed that radiation cooling occurs when the absorption becomes significant in the range of 7 to 14 μm for an optical window in ambient air [51]. For both radiation cooling and radiation heating, it is required to precisely control the absorption resonance wavelength at the MIR spectral range. It is also essential to realize a large area fabrication for most of the practical applications in radiative cooling/heating. The EB-drawing and projection lithography by a stepper reduction of the pattern are promising for precise size control and proof of the principle demonstration. However, the initial and running costs are high for such high-resolution lithographies rendering them not practical at the current stage.

**Figure 6:**
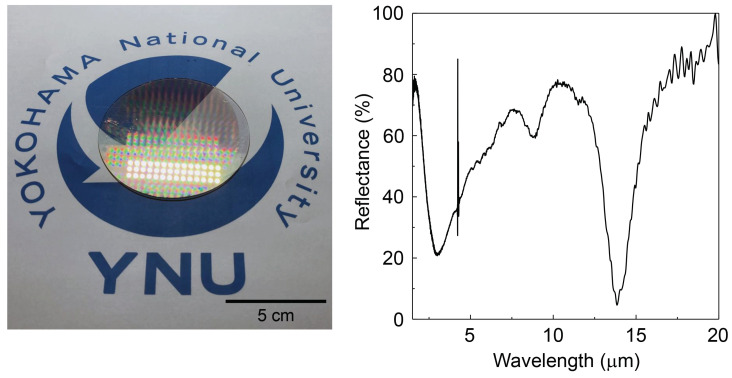
Radiative cooling. Large area fabrication of transparent metasurfaces on a 3-inch diameter MIM (ITO/CaF_2_/Au-micro discs) wafer and its reflectance *R* spectrum. The ∼80% dip in *R* at ∼13 μm is engineered using MIM metasurface fabricated by a simplified photo-lithography approach. Such a window will facilitate radiative heat removal out of the room into air ambient outside. At the visible spectral range, such a window is reflective (see a daylight lamp reflection in the photo). Structure of MIM-wafer: on soda-lime glass, 180 nm of ITO base-layer, CaF_2_ insulator (200 nm), and Au micro-discs on top (50 nm thickness); 2 nm of Cr was used as an adhesion layer.

The contact photo-lithography is the way to reduce the cost of the fabrication and can define structures down to ∼2 μm resolution. Figure 6 shows a large area fabrication of 5 μm Au-disc diameter patterns over an entire 3-inch diameter soda-lime glass wafer with 180 nm ITO film on top. As the insulator layer in the MIM metasurface, a layer of CaF_2_ (200 nm) was used to realize effective absorption in the MIR range. The MIM pattern and its optical properties were well defined over the entire wafer area and showed engineered absorption at around 13 μm wavelength (an ∼80% dip in reflectance). This fabrication method is a step forward for the practical realization of MIR metasurfaces for optical window applications in air ambient. This technique will help future radiation cooling and heating experiments using different materials with aim at reduced cost and lower cost materials made of high abundance elements.

## 4. Conclusions

In this study, we have realized MIM metasurfaces with visible transparency and MIR absorption (emission) for potential applications in the control of thermal radiation. Metasurfaces with ITO substrate reached 80% transparency at visible wavelengths, and a transmission band into the IR-transparency window of air (or other ambient) can be engineered by the choice of MIM pattern. Such ITO-base MIM metasurfaces placed on MIR-transparent windows can be used for radiative cooling at the designed specific spectral range (see the concept in Figure 1). The application of radiative heating and cooling of rooms in the buildings is now undergoing active research for the reduction of electric energy consumption [52] and agriculture greenhouse applications for sustainable energy development.

## Data Availability

Datasets related to this article can be found at http://dx.doi.org/10.33774/chemrxiv-2021-bh4vn, an open-source online data repository hosted at ChemRxiv (last access date 4 October 2022).

## Supplementary Materials

The following supporting information can be downloaded at: https://www.mdpi.com/article/10.3390/mi13101769/s1, Figure S1: Influence of an adhesion nano-layer; Figure S2: Optical permittivities; Figure S3: FDTD simulation with 3D conductive; Figure S4: Conductivity dependencies of FDTD simulations. Figure S5: A series of FDTD simulations for the plasma model with *ε*_∞_= 4.4, *ω*_p_ was changing from 1.0 × 10^14^ to 1.0 × 10^16^ rad/s, Γ is from 1.0 × 10^14^ to 1.0 × 10^16^ 1/s.
