# Supplementary material for: Metasurfaces as Energy Valves for Sustainable Energy Management
Source: Micromachines (Basel). 2022 Oct 18;13(10):1769. doi: 10.3390/mi13101769 (PMC9609501; doi:10.3390/mi13101769)
Supplement: Supplementary file 1 [file micromachines-13-01769-s001.zip › micromachines-1953264-supplementary.pdf]

Supporting information

Metasurfaces as energy valves for sustainable energy management

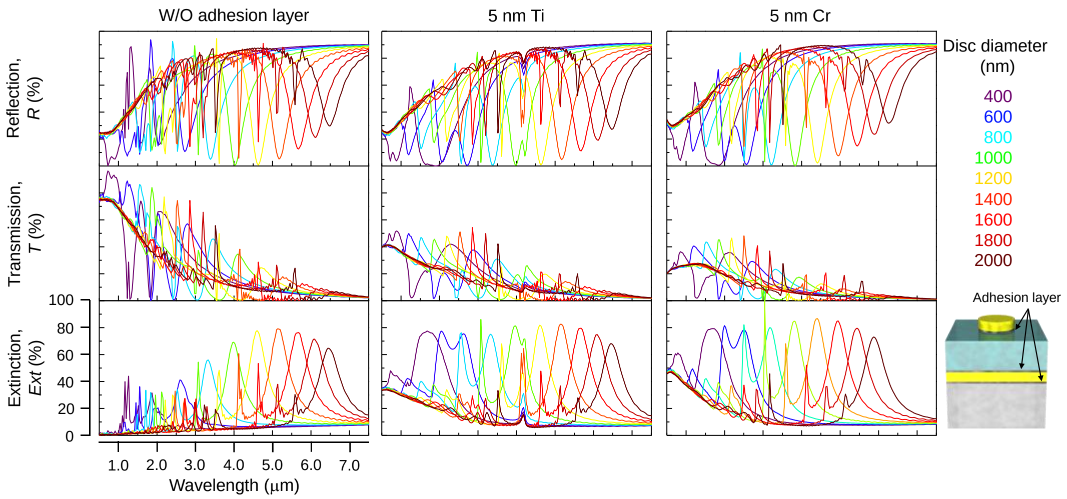

Figure S1 Influence of an adhesion nano-layer. FDTD simulations for thin-Au MIM metasurfaces without adhesion layer, with 5 nm Ti and 5 nm Cr as adhesion layers. Calculations are made for the MIM structure only without substrate.

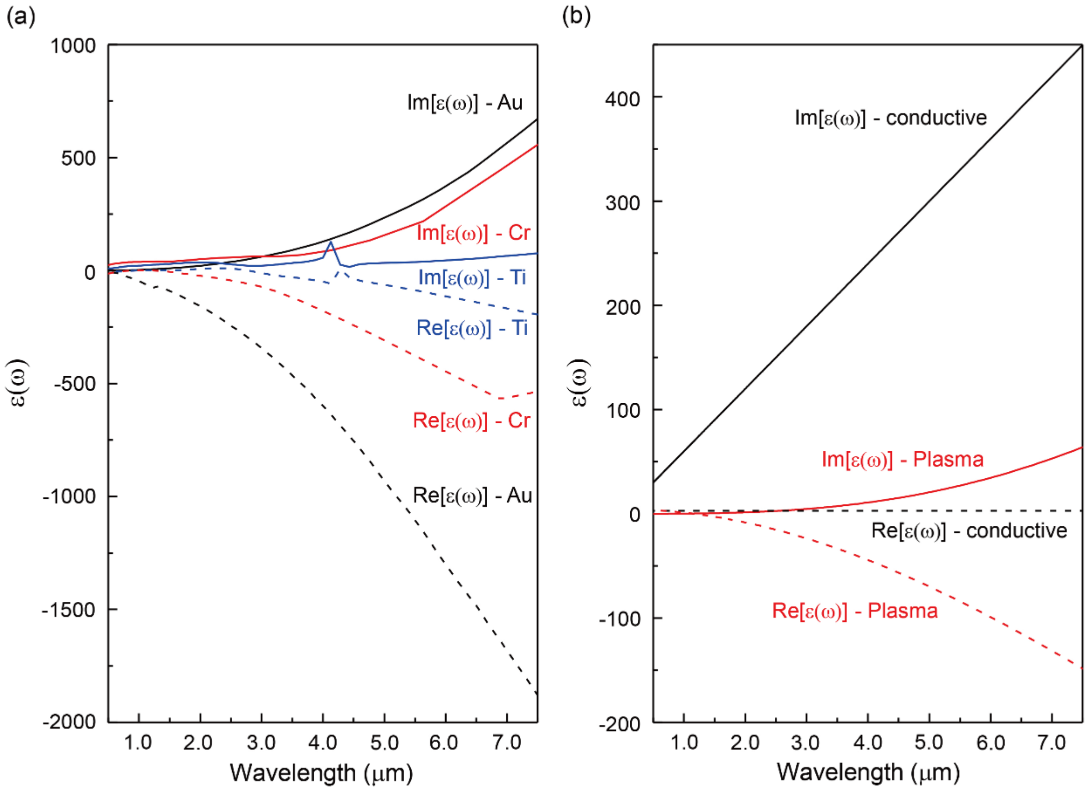

Figure S2 Optical permittivity of materials used in FDTD simulations: (a) Au, Cr, Ti obtained from Palik's database references. (b) Basic function implemented in the FDTD software for the 3D conductive and plasma (Drude) models with parameters of $\varepsilon_{DC} = 2.94$, $\sigma = 1.0\times10^{6}\ (\Omega\text{cm})^{-1}$, $\varepsilon_{\infty} = 4.4$, $\omega_{P} = 3.36\times10^{15}$ rad/s, $\Gamma = 1.05\times10^{14}$ 1/s.

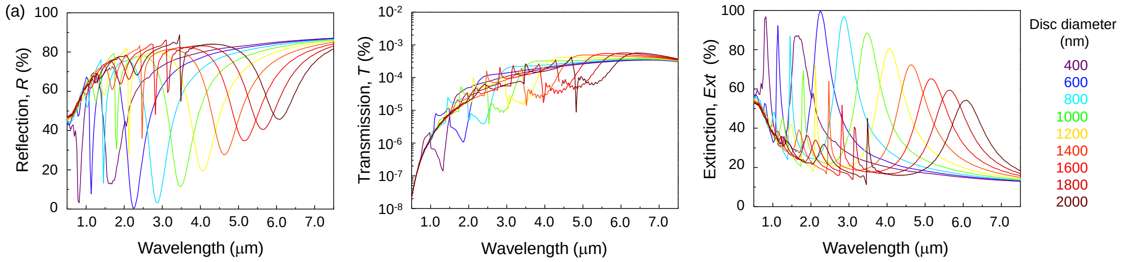

Figure S3 Reflectance R, Transmittance T and Extinction $Ext = 1-(R+T)$ spectra. FDTD simulations for ITO metasurfaces with 3D conductive model with $\varepsilon_{DC} = 2.94$, $\sigma = 1.0\times10^{6}$ $(\Omega\text{cm})^{-1}$.

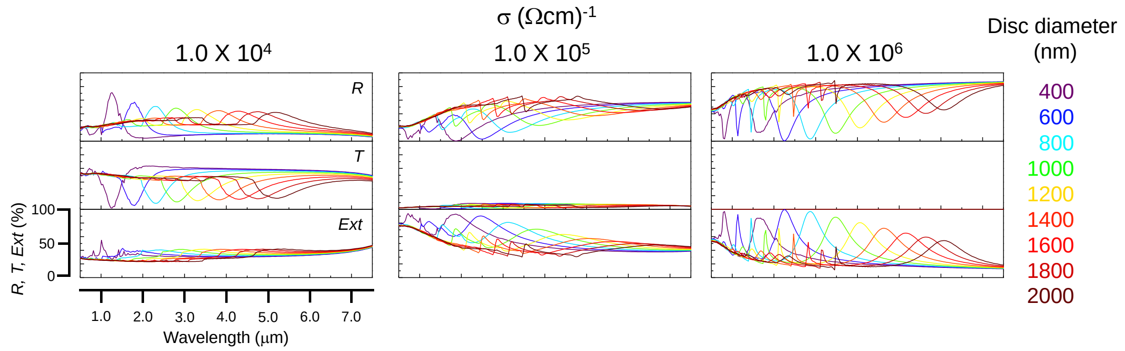

Figure S4 FDTD simulations for the 3D conductive model with $\varepsilon_{DC} = 2.94$ and σ is from 1.0×10^{4} to 1.0×10^{6} $(\Omega\text{cm})^{-1}$.

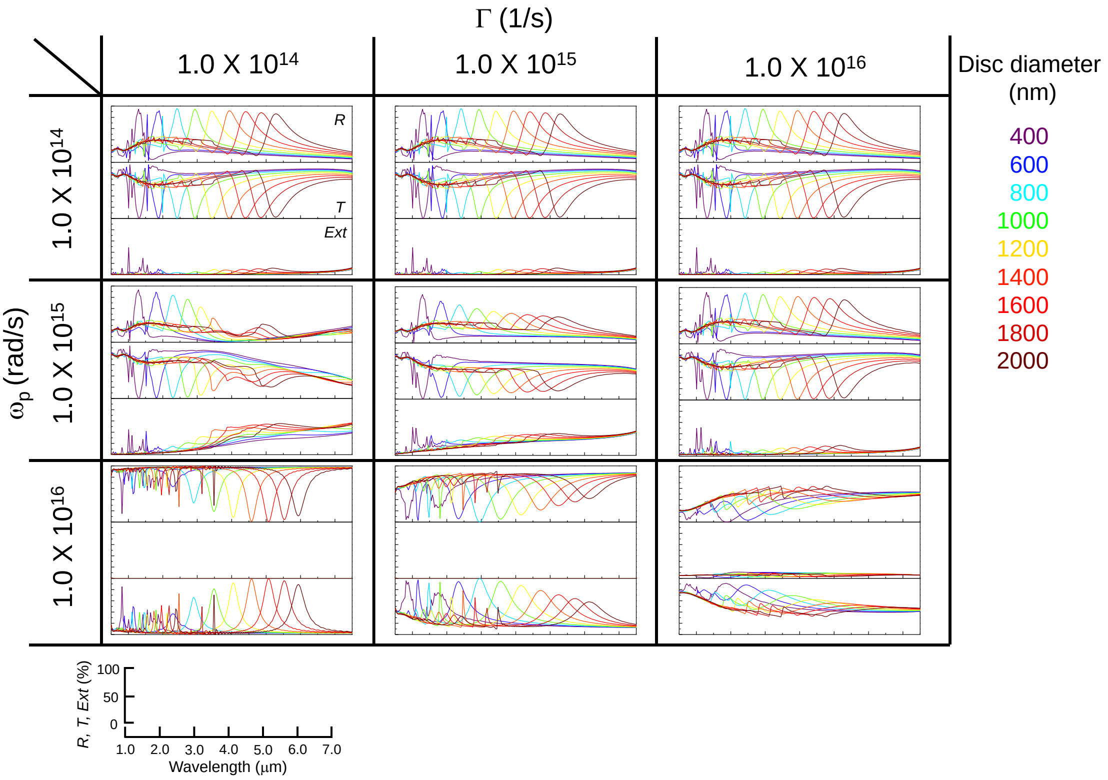

Figure S5 A series of FDTD simulations for the plasma model with $\varepsilon_{\infty} = 4.4$, ω_p was changing from 1.0×10^{14} to 1.0×10^{16} rad/s, Γ is from 1.0×10^{14} to 1.0×10^{16} 1/s.
